# Mental health across the early years in the military

**DOI:** 10.1017/S0033291722000332

**Published:** 2023-06

**Authors:** Lisa Dell, Carolina Casetta, Helen Benassi, Sean Cowlishaw, James Agathos, Meaghan O'Donnell, Monique Crane, Virginia Lewis, Belinda Pacella, Sonia Terhaag, David Morton, Alexander McFarlane, Richard Bryant, David Forbes

**Affiliations:** 1 Phoenix Australia Centre for Posttraumatic Mental Health, 161 Barry Street, Carlton VIC, Melbourne 3053, Australia; 2 Department of Psychiatry, Faculty of Medicine, Dentistry, and Health Sciences, University of Melbourne, Parkville VIC, Melbourne 3053, Australia; 3 Department of Defence, Joint Health Command, Canberra, Australia; 4 Research School of Population Health, Australian National University, Canberra, Australia; 5 Department of Psychology, Faculty of Medicine, Health and Human Sciences, Macquarie University, Macquarie Park NSW, Sydney 2109, Australia; 6 Australian Institute for Primary Care and Ageing, La Trobe University, Bundoora VIC, Melbourne 3086, Australia; 7 Faculty of Health and Medical Sciences, Adelaide Medical School, University of Adelaide, Adelaide SA 5005, Australia; 8 Faculty of Science, School of Psychology, University of New South Wales, Sydney NSW 2052, Australia

**Keywords:** Military, resilience, mental health, PTSD, psychological distress, latent class growth analysis

## Abstract

**Background:**

The mental health impact of the initial years of military service is an under-researched area. This study is the first to explore mental health trajectories and associated predictors in military members across the first 3–4 years of their career to provide evidence to inform early interventions.

**Methods:**

This prospective cohort study surveyed Australian Defence personnel (*n* = 5329) at four time-points across their early military career. Core outcomes were psychological distress (K10+) and posttraumatic stress symptoms [four-item PTSD Checklist (PCL-4)] with intra-individual, organizational and event-related trajectory predictors. Latent class growth analyses (LCGAs) identified subgroups within the sample that followed similar longitudinal trajectories for these outcomes, while conditional LCGAs examined the variables that influenced patterns of mental health.

**Results:**

Three clear trajectories emerged for psychological distress: resilient (84.0%), worsening (9.6%) and recovery (6.5%). Four trajectories emerged for post-traumatic stress, including resilient (82.5%), recovery (9.6%), worsening (5.8%) and chronic subthreshold (2.3%) trajectories. Across both outcomes, prior trauma exposure alongside modifiable factors, such as maladaptive coping styles, and increased anger and sleep difficulties were associated with the worsening and chronic subthreshold trajectories, whilst members in the resilient trajectories were more likely to be male, report increased social support from family/friends and Australian Defence Force (ADF) sources, and use adaptive coping styles.

**Conclusions:**

The emergence of symptoms of mental health problems occurs early in the military lifecycle for a significant proportion of individuals. Modifiable factors associated with wellbeing identified in this study are ideal targets for intervention, and should be embedded and consolidated throughout the military career.

## Introduction

Military life can be extremely rewarding but, for some, equally challenging. A significant body of literature exists informing the impact of military experiences during/after deployment and transition out of the military (Cheok, Ang, Chew, & Tan, 2000; Mobbs & Bonanno, 2018); however, there is a critical gap in understanding the health and wellbeing impacts of initial enculturation into the military. The first few years of a military career encompass initial training through to advanced training and into postings and eventually to readiness for deployment and represents a unique period of time in a military career – one that is unlikely to be repeated. Understanding what influences wellbeing over this unique period, the risks and protective factors, is crucial for maintaining a healthy and ready workforce. This study is the first to identify these key factors and provides an early look at mental health trajectories across the initial years of a military career, offering an important opportunity to pinpoint how and when to build resilience and mitigate the development of psychiatric disorder in Defence forces.

Longitudinal studies with military populations have characterized trajectories of post-traumatic stress symptoms following deployment or transition out of the military (Andersen, Karstoft, Bertelsen, & Madsen, 2014; Berntsen et al., 2012; Boasso, Steenkamp, Nash, Larson, & Litz, 2015; Bonanno et al., 2012; Dickstein, Suvak, Litz, & Adler, 2010; Eekhout, Reijnen, Vermetten, & Geuze, 2016; Karsten, Penninx, Verboom, Nolen, & Hartman, 2013; Nash et al., 2015; Orcutt, Erickson, & Wolfe, 2004). These studies provide unique and valuable insights about changes in mental health over time, and provide the opportunity to explore factors that predict such changes. Historically, trajectory studies on military samples have identified three to six classes or profiles. Typically, military personnel demonstrate stable, low levels of post-traumatic stress symptoms following deployment (the *resilient* class); however, three other classes are also commonly found: a *chronic* class (high, stable symptoms indicated of disorder), a *worsening* class (mild/moderate symptoms that escalate to disorder) and a *recovered* class (mild/moderate symptoms that reduce to low symptoms). Additional classes for moderate or subthreshold symptom levels have been identified, including a *temporary-benefit* and *pre-existing symptom* class of high symptoms that initially decrease, but remain fluctuating in the subthreshold zone over time (Andersen et al., 2014; Berntsen et al., 2012; Nash et al., 2015; Orcutt et al., 2004). Although comparatively far less literature has examined symptom trajectory heterogeneity for general psychological distress in military populations, the same core trajectory classes appear to be relevant, with evidence supporting the existence of *resilient*, *recovery* and *worsening* classes (Cabrera & Adler, 2021; Palmer et al., 2019).

To date, no studies have examined mental health trajectories across the initial years of the military career, and whether comparable classes emerge at this point in time is unknown. The early period of a military career will bear its own unique challenges for individuals, and whilst the transitions and adjustments are markedly different to those during deployments or transitioning out of service, the stress and adjustment associated with integrating into military life will be burdensome for some. The extent to which this stress is reflected in mental health symptoms is currently unknown. Addressing this gap is vital in developing our ability to identify those at risk of developing psychopathology over the military career, and to inform the development of early resilience training and at what time-points in order to promote protective mental health factors.

Previous studies have provided insight into predictors of adjustment to the military, showing that most military members adjust well but some experience periods of increased stress and adversity, varying in levels of severity from subthreshold adjustment difficulties to full psychiatric disorder (Booth, Probert, Forbes-Ewan, & Coad, 2006; Cheok et al., 2000; Nakkas, Annen, & Brand, 2016). Conscientiousness, emotion regulation, social support and impulse control are factors that have been shown to promote psychological adjustment to military life within the first 6 years of voluntary (Lee, Sudom, & Zamorski, 2013; Sudom, Lee, & Zamorski, 2014) and compulsory (Choi et al., 2012; Kim et al., 2015) basic training and service, whereas exposure to early life stress, and difficulties with military peers and superiors can lead to maladjustment (Cheok et al., 2000; Choi et al., 2012). Integrating into military culture involves significant cultural, physical, psychological and occupational changes, including moving away from established support networks, complying with military protocols, values and ethics, and changing sleeping patterns (Martin, Williamson, Alfonso, & Ryan, 2006; Mobbs & Bonanno, 2018). This indicates there may be unique individual and/or situational protective and risk factors that influence whether service members adjust or experience increased stress and potentially psychological maladjustment. In order to build on this extant literature, we need to move beyond cross-sectional studies to follow a consistent cohort, tracking patterns and changes in mental health and wellbeing and to identify specific factors and for whom that may promote or erode adjustment in the initial years of service and the risks this poses for further exacerbation of mental health status.

Knowing that the early career period of military service exposes members to unique stressors (e.g. moving away from established supports, rigorous training, handling weapons, changes in sleep) and challenges (Booth et al., 2006; Cheok et al., 2000; Nakkas et al., 2016), the purpose of this study is to explore the mental health trajectories, and subsequent predictors of psychological resilience and vulnerability as highlighted in the extant literature, for the first time. This study is an invaluable contribution to our understanding of how to promote wellbeing and mitigate the development of mental health symptoms or disorder in military personnel from the very outset of a military career.

This study utilized data from a nationally representative, prospective cohort study of 5329 Australian Defence Force (ADF) members. This study aimed to (1) identify the trajectories of key mental health outcomes over the first 3–4 years of a military career and (2) identify factors, many potentially modifiable, associated with these trajectories during these early years.

## Methods

### Study design and participants

Data were drawn from the Longitudinal ADF Study Evaluating Resilience (LASER-Resilience), a nationally representative, prospective cohort study. The study used a phased enrolment strategy with a sampling frame of all new full-time general entry (GE) enlistees with surnames between *L*–*Z* and all appointed officers entering the Australian Navy, Army or Air Force between November 2009 and December 2012 (participant *n* = 5329). All newly enlisted GEs with surnames beginning with the letters L–Z were eligible for inclusion. To avoid over-surveying members in the ADF, those with surnames commencing A–K were instead recruited into a separate study that was being conducted concurrently. Previous analyses (Crane et al., 2012) have confirmed that there were no systematic differences in common baseline measures between the two groups of GEs (i.e. A–K and L–Z). Given that officer appointees comprised a much smaller population than GEs, there were concerns about dividing this sample and reducing the capacity to examine officers as a unique sub-population; therefore, all officer appointees were eligible for this study.

Participants were surveyed over five time points from enlistment/appointment to 4 years post-enlistment/appointment between November 2009 and November 2016. T1 data collection occurred at the point of enlistment for GEs, and within the first few weeks of training commencement for Officers. For most participants, T2 occurred at the completion of initial training (with Officer Cadets completing T2 12 months into initial training). At the conclusion of participants' subsequent employment training, which qualifies individuals for their chosen profession within the Armed Forces, participants completed the T3 assessment, which was approximately one year after T2, and before entering their first posting. Subsequent time points of data collection occurred annually after completion of T3, at T4 and T5. There were a total of *n* = 5696 survey responses at T1, and *n* = 5329 participants also returned surveys at T2. Subsequently, there were *n* = 1759 participants that returned surveys at T3 and could be linked with T2 responses, while comparable numbers were *n* = 1271 at T4, and *n* = 1194 at T5. Across the latter time-points, there were also surveys that could not be linked with earlier survey records (T3: *n* = 552; T4: *n* = 497; and T5: *n* = 456). Accordingly, these unlinked surveys were not informative for purposes of prospective analyses, and are not considered further here. Preliminary analyses of the analytic sample (comprising surveys that could be linked prospectively over time) revealed limited evidence of systematic bias from study attrition (see online Supplementary Table S1). Questionnaires were administered in paper form at T1 and T2 and from T3 onwards were administered online using the surveying tool Opinio (Version 6.3.3). Due to the current study's focus on early career military service, we examined data collected from T2 to T5. T2 was selected as the most informative ‘baseline’ for these analyses as this marked the post-initial training period, when participants had progressed to being part of the ‘trained force’. All participants in the LASER-Resilience study who completed assessments at T2 (*n* = 5329) were consequently included for analysis. See online Supplementary Fig. S2 for a summary of data collection points throughout the study. The study was approved by the Australian Defence Human Research Ethics Committee (protocol number: 556-09).

### Measures

The primary outcome measures were *psychological distress*, measured using the 10-item K10 (Schuster, Kessler, & Aseltine, 1990) and *posttraumatic stress symptomatology* measured via the four-item PTSD Checklist (PCL-4), a shortened version of the original PCL-C (Weather, Litz, Herman, Huska, & Keane, 1993). Total scores are calculated for both core outcomes by summing items to produce a summed score – possible scores ranged from 10–50 on the K10 and 4–20 on the PCL-4, with higher scores signifying higher symptom severity.

Predictor variables included sociodemographic measures (see Table 1) and service-related factors (rank and ADF service branch). Additional predictors included *social support*; measured via a modified version of the ‘supportive and negative social interactions’ scale (Schuster et al., 1990) which separately measures the frequency of positive and negative social interactions across two subscales (modified for the military context by separately examining colleague and leadership interactions, thus forming four subscales); *exposure to potentially traumatic events* was examined from T2 onwards using a 15-item Potentially Traumatic Life Events Checklist, quantified as the aggregate number of potentially traumatic events experienced; perceived *sleep impairment* was measured via a modified four-item version of the Sleep Impairment Index (SII; retaining items assessing difficulty falling asleep and staying asleep, problems waking too early and satisfaction with current sleeping patterns), where total summed scores range from 0 to 24, with higher scores indicating greater sleep impairment; *anger* was measured via the five-item Dimensions of Anger Reactions (DAR-5) (Forbes et al., 2014, 2004; Hawthorne, Mouthaan, Forbes, & Novaco, 2006), where total summed scores range from 5 to 25, with higher scores indicating greater severity of anger symptomatology; *coping styles* were assessed by an adapted 24-item version of the 28-item ‘Brief COPE’ inventory (Carver, 1997). Based on previous analyses (Crane, Kehoe, Reid, & Casetta, 2012), 17 items from the full scale were grouped to form six coping style variables. One item from the reappraisal scale was removed from analysis due to problematic psychometric properties. There was low inter-item correlation (>0.15) within the avoidance and risk-taking subscales, so a single item with the greatest face validity was used for each coping style (‘*I avoid thinking or talking about the situation*’ and ‘*I engage in risk-taking behaviour, such as speeding, drinking too much or risky sexual behaviour*’ respectively). As such, the final measure comprised the acceptance (two items), reappraisal (two items), self-blame (two items), avoidance (one item), risk-taking (one item) and support-seeking (four items) subscales. Scores for each subscale were calculated by summing the scores for each item, which were measured on a four-point Likert scale describing the frequency of use for each style (1 = not at all, 4 = a lot).

**Table 1.** Sociodemographic characteristics of the sample at T2 (*n* and %)^a^

	T2
	*N* (%)
Age, years – mean (s.d.)	22.19 (5.17)
Gender	
Male	4527 (85.6%)
Female	763 (14.4%)
Relationship status	
Single/never married	3730 (70.4%)
Married/partner	1514 (28.6%)
Divorced/separated/widowed	55 (1.0%)
Education	
Completed year 10	440 (12.7%)^b^
Completed year 12	2070 (59.9%)^b^
Post school training	477 (13.8%)^b^
Tertiary qualification	470 (13.6%)^b^
ADF service	
Army	3351 (63.1%)
Navy	1145 (21.6%)
Air Force	814 (15.3%)
Rank	
Officers	1964 (36.9%)
Other rank	3288 (61.7%)

a Note that T2 marked end of initial training, at which point no participants had been deployed. At subsequent time points, deployment rates ranged from <7% at T3 to 20% at T5.

b Measured at T1.

Preliminary analyses of scale unidimensionality and internal reliability suggested all final scales had strong psychometric properties, with strong evidence to support the usage of a single summed scale score (see online Supplementary Table S3).

### Data analysis

Analysis were conducted in MPlus version 8 (Muthén & Muthén, 2017), and used full information maximum likelihood estimation to utilize all available data. Latent class growth analyses (LCGAs) models were estimated for the two primary outcomes to identify homogenous subgroups within the sample that followed similar trajectories over time. An exploratory approach was adopted to identify the optimal number of latent classes, and initially involved the estimation and comparison of models specifying one through five classes. These were estimated using MLR estimation and were initially compared using statistical indices. These included the Akaike Information Criteria (AIC) (Akaike, 1998) and Bayesian Information Criteria (BIC) (Schwarz, 1978), for which lower values indicate better model fit, as well as the Lo-Mendell-Rubin Likelihood Ratio Test (LMR LRT), which provides a direct significance test of the relative improvement in fit for two nested models. The entropy statistic was also considered and indicates the classification accuracy of different class models. Entropy values range from 0.0 to 1.0 and estimates >0.70 are classified as acceptable. Once models that specified the provisional number of latent profiles were determined, graphical tools (depicting the observed/estimated means and trajectories for each class) were produced to help interpret classes and inform final decisions about the suitability of the final class models.

Conditional LCGAs were then specified in order to examine the individual and situational variables that were associated with the patterns of mental health (as identified by unconditional LCGA models for the K10 and PCL-4) in the context of stressors. These analyses considered predictor variables that were measured at T2, including sociodemographic characteristics, service-related variables, as well as social support, total number of lifetime PTEs, coping styles, anger and sleep impairment.

For each outcome variable, predictors were considered separately in a series of ‘bivariate’ analyses comprising conditional LCGA models that evaluated the predictors of ‘class membership’ using the three-step procedure in MPlus. The latter analyses are equivalent to multinomial logistic models in which class membership is regressed on the T2 explanatory variable, producing effects comparing each class with a ‘reference category’. Given that the preferred LCGA models for each outcome identified a large class defined by consistently low scores over time, this was specified as the reference category of greatest interest for purposes of interpretation.

## Results

Key sociodemographic characteristics of the sample at T2 are presented in Table 1.

Approximately 70% of participants reported some level of lifetime exposure to potentially traumatic events at T2, most frequently involving witnessing someone being badly injured or killed, natural disasters, and being threatened without a weapon (see Table 2).

**Table 2.** Prevalence of lifetime potentially traumatic events

Traumatic event	T2
	*N* (%)
Direct combat	458 (8.8%)
Life-threatening accident	1155 (22.1%)
Fire, flood or other natural disaster	1650 (31.5%)
Witness someone badly injured or killed	1767 (33.8%)
Sexual molestation	118 (2.3%)
Serious physical attack or assault	1270 (24.2%)
Threatened with a weapon/held captive/kidnapped	574 (11.0%)
Tortured or victim of terrorists	23 (0.4%)
Domestic violence	436 (8.3%)
Witness domestic violence	931 (17.8%)
Finding dead body	394 (7.5%)
Child abuse – physical	204 (3.9%)
Child abuse – emotional	291 (5.6%)
Rape	79 (1.5%)
Threatened/harassed without a weapon	1671 (31.9%)
Witness someone suicide or attempt suicide	588 (11.2%)
Suffer great shock event to someone close	642 (12.6%)
Any other event	230 (5.0%)
**Any past event**	**3686** (**70.2%)**

**Fig. 1.**
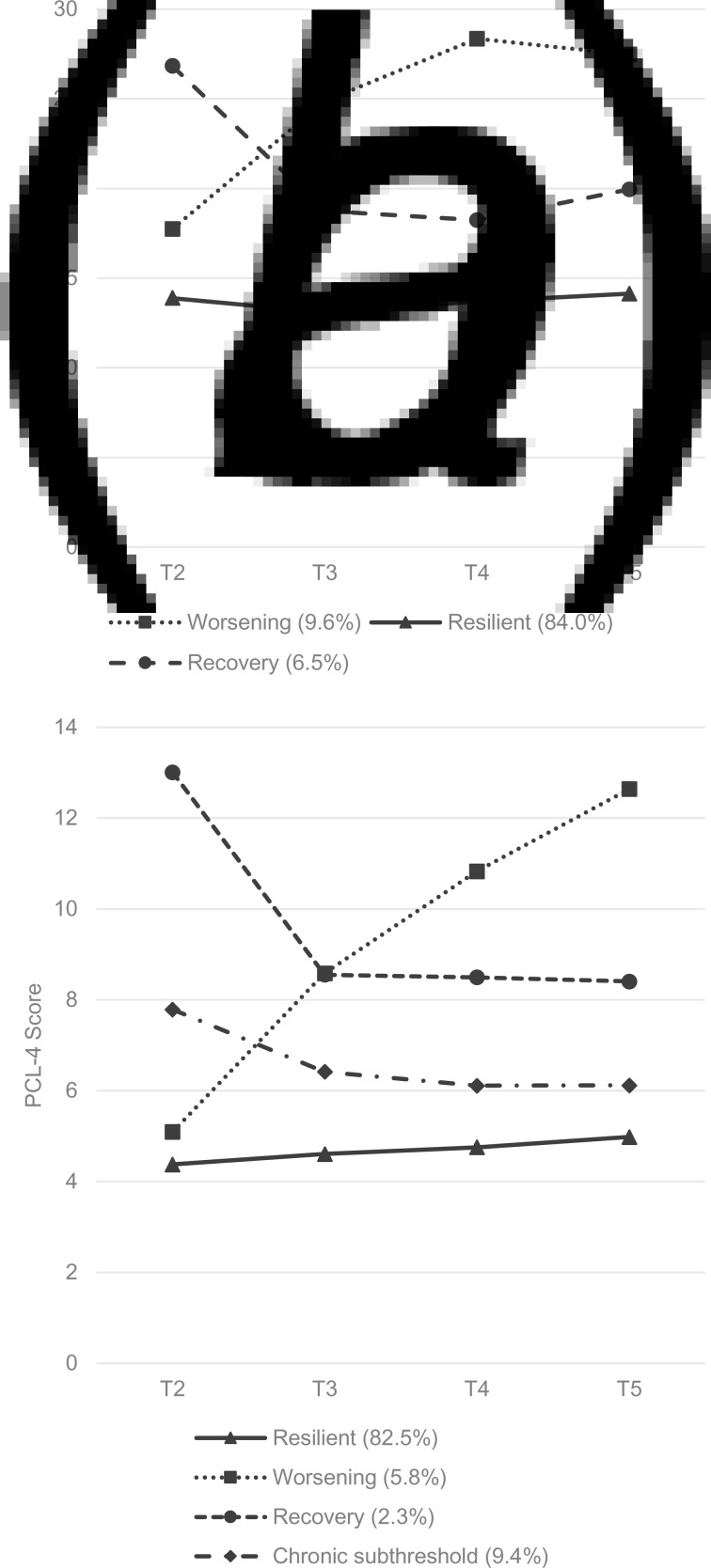
Class-specific mean trajectories over time for (*a*) three-class model of K10 scores, and (*b*) four-class model of PCL-4 scores.

Fit indices from a series of potentially plausible unconditional LCGAs were estimated for the K10 (see online Supplementary Table S4). While the AIC and BIC values indicated improvements across increasingly complex models based on the K10, the LMR-LRT suggested no significant improvement in fit between the three-class and four-class models. Accordingly, the three-class model provided the most suitable account of subpopulations underlying the trajectories of K10 scores. These included (1) low and stable class, or ‘resilient’ (84.0%); (2) low and increasing class, ‘worsening’ (9.6%); and (3) high and decreasing class, ‘recovery’ (6.5%). Class-specific mean trajectories for the K10 are presented in Fig. 1*a*.

Fit indices from unconditional LCGAs were estimated for the PCL-4 (see online Supplementary Table S4), and these indicated that two-class, three-class and four-class solutions were all statistically plausible descriptions of trajectories underlying PCL-4 scores (in contrast, the LMR-LRT indicated no significant difference between the four-class and five-class solutions). The four-class solution was selected as the preferred model given substantive interest in the specific groups identified by this class model. These included (1) resilient class (82.5%); (2) worsening class (5.8%); (3) recovery class (2.3%); and (4) moderate and decreasing class, ‘chronic-subthreshold’ (9.4%). Figure 1*b* provides a graphical depiction of the class-specific mean trajectories for the PCL-4.

Table 3 shows results for multinomial regression analyses comparing the worsening and resilient classes for psychological distress. Being male was the strongest predictor of belonging to the resilient group, relative to the group that demonstrated worsening distress symptoms over time. Participants reporting high levels of social support from family/friends, ADF peers and ADF superiors, as well as those more frequently using acceptance and reappraisal coping styles were also more likely to be in the resilient group. Conversely, the use of self-blame and risk-taking coping styles was most strongly associated with decreased likelihood of being resilient and increased likelihood of demonstrating worsening symptoms over time; while high trauma exposure; negative social interactions with family/friends, ADF peers and ADF superiors; more frequent use of avoidance as a coping style; high levels of anger; and sleep problems were also key predictors.

**Table 3.** Conditional LCGA models with T2 predictors of class membership for the preferred three-class model of K10 scores

	Resilient *v*. worsening				Resilient *v*. recovery			
	Estimate	s.e.	Odds ratio	95% CI	Estimate	s.e.	Odds ratio	95% CI
Age	−0.02	0.02	0.98	0.95–1.01	−0.03	0.01	0.97*	0.95–1.00
Gender (ref: female)								
Male	−0.58	0.20	0.56**	0.38–0.84	−0.59	0.16	0.55**	0.40–0.76
Relationship status (ref: single)								
Partnered/div/sep/wid	−0.22	0.19	0.81	0.56–1.16	−0.05	0.15	0.95	0.71–1.26
Number of children	0.06	0.12	1.06	0.84–1.34	0.03	0.12	1.03	0.82–1.29
Rank (ref: GE)								
Officer	0.01	0.17	1.01	0.72–1.40	−0.17	0.14	0.85	0.65–1.10
ADF service (ref: Army)								
Navy	0.21	0.20	1.24	0.84–1.82	0.34	0.15	1.41*	1.04–1.91
Air Force	−0.09	0.24	0.92	0.57–1.48	−0.01	0.19	0.99	0.68–1.45
Social support								
Family/friend social support	−0.30	0.05	0.74***	0.67–0.81	−0.38	0.03	0.68***	0.64–0.73
ADF social support								
Peer social support	−0.38	0.07	0.68***	0.60–0.78	−0.57	0.05	0.57***	0.51–0.62
Superior social support	−0.18	0.06	0.8**	0.75–0.93	−0.42	0.04	0.66***	0.60–0.71
Negative social interactions								
Family/friend	0.21	0.04	1.23***	1.15–1.32	0.20	0.03	1.22***	1.16–1.29
ADF peers	0.27	0.05	1.31***	1.20–1.43	0.47	0.04	1.60***	1.47–1.73
ADF superiors	0.17	0.04	1.19***	1.10–1.28	0.37	0.03	1.45***	1.36–1.55
Number of traumatic events	0.11	0.03	1.12**	1.05–1.19	0.15	0.02	1.16***	1.11–1.21
Coping styles								
Acceptance	−0.20	0.07	0.82**	0.72–0.93	−0.65	0.05	0.52***	0.47–0.58
Reappraisal	−0.16	0.06	0.85**	0.76–0.96	−0.49	0.05	0.61***	0.56–0.68
Self-blame	0.41	0.05	1.51***	1.36–1.67	0.74	0.05	2.09***	1.91–2.29
Avoidance	0.21	0.09	1.24*	1.04–1.47	0.58	0.07	1.79***	1.55–2.06
Risk-taking	0.40	0.11	1.49***	1.2–1.86	0.68	0.08	1.97***	1.7–2.29
Support-seeking	−0.05	0.03	0.95	0.9–1.01	−0.06	0.03	0.94*	0.89–0.99
Anger	0.20	0.02	1.22***	1.17–1.26	0.25	0.02	1.29***	1.25–1.32
Sleep problems	0.29	0.03	1.34***	1.26–1.42	0.35	0.02	1.59***	1.52–1.67

CI, confidence interval. The low symptom (resilience) class served as the reference group. *p ⩽ 0.05, **p ⩽ 0.01, ***p ⩽ 0.001.

Table 3 also shows comparisons between the recovery and the resilient classes. While the resilient group displayed consistently low symptom severity over time, the recovery group was characterized by initially high symptoms which decreased over time. Participants who were male; reported high social support from family/friends, ADF peers and ADF superiors; and used acceptance and reappraisal coping styles were less likely to belong to the recovery group, relative to the resilient category – being male was again the strongest predictor of belonging to the resilient group. Participants who reported more traumatic events; negative social interactions from family/friends, ADF peers and superiors; more frequent use of self-blame, avoidance and risk-taking coping styles; as well as anger and sleep problems, were more likely to belong to the recovery group; the use of self-blame, risk-taking and avoidance coping styles were particularly strongly associated with the recovery trajectory. Further comparisons between other classes on the K10 outcome are depicted in online Supplementary Table S5.

Table 4 shows results from conditional LCGA models that specified predictor variables at T2 in the preferred four-class model of PCL-4 trajectories. Results from multinomial regression analyses comparing the worsening and resilient classes indicated that males and Officers were far more likely to be in the resilient group rather than the group with worsening symptoms, while participants reporting high social support from family/friends and ADF peers/superiors were also more likely to belong to the resilient group. Conversely, Navy service (relative to Army), number of traumatic events, use of self-blame coping, anger and sleep problems were all associated with increased likelihood of belonging to the worsening class, with Navy service acting as the strongest predictor. Further comparisons between other classes on the PCL-4 outcome are depicted in online Supplementary Table S6.

**Table 4.** Conditional LCGA models with T2 predictors of class membership for the preferred four-class model of PCL-4 scores

	Resilient *v*. recovery				Resilient *v*. chronic-subthreshold				Resilient *v*. worsening			
	Estimate	s.e.	OR	95% CI	Estimate	s.e.	OR	95% CI	Estimate	s.e.	OR	95% CI
Age	−0.01	0.02	0.99	0.96–1.02	−0.03	0.01	0.97*	0.95–1.00	0.03	0.02	1.03*	1.00–1.06
Gender (ref: female)												
Male	−0.68	0.23	0.51**	0.33–0.79	−0.37	0.14	0.69**	0.53–0.90	−1.08	0.23	0.34***	0.22–0.53
Relationship (ref: single)												
Partnered/DSW	−0.13	0.21	0.88	0.58–1.32	−0.12	0.11	0.89	0.71–1.11	0.13	0.21	1.14	0.75–1.73
Number of children	0.01	0.15	1.01	0.75–1.35	−0.05	0.09	0.95	0.79–1.14	0.09	0.14	1.09	0.82–1.45
Rank (ref: GE)												
Officer	−0.66	0.19	0.52***	0.36–0.74	−0.35	0.10	0.71**	0.58–0.87	−0.48	0.20	0.62*	0.42–0.91
ADF service (ref: Army)												
Navy	0.42	0.21	1.52*	1.01–2.29	0.19	0.12	1.21	0.95–1.53	0.53	0.23	1.69*	1.07–2.67
Air Force	−0.33	0.31	0.72	0.39–1.32	−0.14	0.15	0.87	0.65–1.18	0.31	0.28	1.36	0.79–2.33
Social support												
Family/friend social support	−0.46	0.05	0.63***	0.58–0.69	−0.27	0.03	0.76***	0.72–0.81	−0.19	0.06	0.83**	0.73–0.94
ADF social support												
Peer social support	−0.68	0.07	0.51***	0.44–0.58	−0.34	0.04	0.71***	0.6–0.77	−0.25	0.08	0.78**	0.67–0.91
Superior social support	−0.43	0.06	0.65***	0.58–0.74	−0.23	0.03	0.80***	0.75–0.85	−0.02	0.07	0.99	0.86–1.13
Negative social interactions												
Family/friend	0.24	0.04	1.28***	1.19–1.37	0.16	0.02	1.18***	1.13–1.23	0.01	0.04	1.01	0.93–1.09
ADF peers	0.41	0.05	1.51***	1.37–1.66	0.26	0.03	1.30***	1.23–1.37	0.10	0.06	1.11	0.99–1.24
ADF superiors	0.35	0.05	1.41***	1.29–1.54	0.19	0.03	1.21***	1.15–1.27	0.03	0.05	1.03	0.94–1.12
Number of traumatic events	0.24	0.03	1.27***	1.2–1.34	0.11	0.02	1.12***	1.07–1.16	0.12	0.04	1.13**	1.05–1.22
Coping styles												
Acceptance	−0.62	0.08	0.54***	0.46–0.62	−0.39	0.04	0.68***	0.62–0.73	−0.08	0.09	0.92	0.77 −1.10
Reappraisal	−0.42	0.07	0.66***	0.58–0.75	−0.31	0.04	0.73***	0.68–0.78	0.01	0.07	1.01	0.88–1.16
Self-blame	0.83	0.06	2.28***	2.03–2.57	0.50	0.03	1.64***	1.54–1.75	0.21	0.07	1.23**	1.08–1.40
Avoidance	0.71	0.11	2.03***	1.63–2.51	0.43	0.06	1.54***	1.39–1.72	−0.17	0.13	0.84	0.66–1.08
Risk-taking	0.84	0.10	2.32***	1.92–2.80	0.50	0.07	1.65***	1.44–1.88	0.29	0.15	1.33	1.00–1.78
Support-seeking	−0.04	0.04	0.96	0.89–1.04	−0.03	0.02	0.97*	0.93–1.00	0.04	0.04	1.04	0.97–1.11
Anger	0.25	0.02	1.28***	1.25–1.32	0.17	0.01	1.19***	1.16–1.21	0.06	0.03	1.06*	1.01–1.12
Sleep problems	0.46	0.03	1.59***	1.50–1.68	0.33	0.02	1.39***	1.34–1.44	0.12	0.04	1.13**	1.05–1.22

OR, odds ratio; CI, confidence interval. *p ⩽ 0.05, **p ⩽ 0.01, ***p ⩽ 0.001.

The low symptom (resilient) class served as the reference group.

Generally similar patterns of association were observed for comparisons between the recovery and resilient categories, except that social support from ADF superiors and both acceptance and reappraisal coping were associated with reduced likelihood of belonging to the recovery category, while the use of avoidance and risk-taking coping styles was also associated with increased likelihood of belonging to this category. Comparable associations were also observed for comparisons between the chronic-subthreshold *v.* resilient classes, except that support-seeking was weakly associated with reduced likelihood of belonging to the chronic-subthreshold category. The magnitude of associations, as indicated by odds ratios, appears larger for the comparisons between the resilient and recovery groups than for the resilience and chronic subthreshold groups. This indicates that the predictor variables more clearly distinguished individuals in the resilient group from those in the recovery group than from those in the chronic subthreshold group.

## Discussion

This study is the first to explore the mental health impact of the early years in the military on its members. It offers a first look into the potentially modifiable factors that can influence the trajectories of mental health and potential mitigation of psychiatric disorder. Interestingly, the trajectories of mental health that emerged in this study and the approximate proportion of members in each were similar to those found in personnel who had deployed or were transitioning out of the military (Andersen et al., 2014; Berntsen et al., 2012; Boasso et al., 2015; Bonanno et al., 2012; Dickstein et al., 2010; Eekhout et al., 2016; Karsten et al., 2013; Nash et al., 2015; Orcutt et al., 2004), indicating that the emergence of mental health symptoms is occurring very early in the lifecycle of a military member, and in the absence of events such as deployment. Three clear trajectories emerged for psychological distress, comprising resilient, recovery and worsening trajectories with four trajectories emerging for post-traumatic stress symptoms; resilient, recovery, worsening and chronic subthreshold trajectories. It is important to note that although a ‘recovery’ trajectory clearly emerged (symbolic of high scores that decreased over time), individuals within this group were still endorsing a number of symptoms of poor mental health and as such they should be considered an at-risk group that is worthy of follow-up and potential intervention.

A number of key factors emerged as predictors of the identified trajectories, similar across both psychological distress and post-traumatic stress symptom trajectories. The patterns of predictor variables largely indicated that prior trauma exposure and critically specific modifiable factors, including the use of maladaptive coping styles (i.e. self-blame, avoidance and risk-taking), negative social interactions, increased anger and sleep difficulties are more closely associated with the worsening and chronic subthreshold groups, whilst members in the resilience trajectories were more likely to be male, report increased social support from family/friends and Defence sources, and use adaptive coping styles (i.e. acceptance, reappraisal). These findings are consistent with previous literature in the early military career period, which indicate that social support (Choi et al., 2012; Kim et al., 2015; Lee et al., 2013; Sudom et al., 2014) and use of adaptive coping styles promote psychological adjustment (Britt, Crane, Hodson, & Adler, 2016; Nakkas et al., 2016), whilst previous traumatic exposure and difficulties with military peers and superiors predict maladjustment (Cheok et al., 2000; Choi et al., 2012). It may be that high levels of unit cohesion and interconnectedness act as a social resource that buffers against unique stressors experienced in the early years of a military career (Mobbs & Bonanno, 2018), and given the low autonomy context of the military, adaptive coping styles such as acceptance may be particularly beneficial (Britt et al., 2016). It is prudent to note that the directionality of the relationship between social support and trauma is complex, with evidence suggesting that greater posttraumatic stress symptoms predict decreased perceived positive social support over time (Nickerson et al., 2017) and as such the causation argument is yet to be clearly defined. Regardless, intervention to strengthen and enhance social supports seems imperative in mitigating and managing the emergence of mental health symptoms. It is additionally notable that membership of the recovery class relative to the resilient class was predicted by several known risk factors, such as maladaptive coping styles and lack of social support – in conjunction with the fact that their symptom severity post-recovery was still above those in the resilient class, this may suggest that that individuals in the recovery class may yet remain at risk of symptom relapse over time.

Strikingly, most of the key predictors associated with poorer mental health trajectories represent modifiable factors (e.g. maladaptive coping strategies, levels of anger and sleep difficulties), and as such, these factors could be targeted via early mental health screening, training and psychological interventions. A viable approach may be to more actively involve leadership and/or command members in managing the wellbeing of their unit, particularly junior leadership. Perceived good leadership has previously been associated with lower levels of mental disorder, including PTSD, in Armed Forces members (Jones et al., 2012) and given the knowledge of personnel in their command that leaders have, they are well-placed to identify changes in an individual'

This study has identified, for the first time, the specific modifiable factors that were associated with poorer mental health trajectories in early career military members. Importantly, there is an existing evidence base to guide support and intervention of these factors. Intervention may include; for sleep problems: Cognitive Behavioural Therapy (CBT) for Insomnia (Troxel et al., 2015); for anger: CBT-based anger management (Cash et al., 2018; Deffenbacher, 1999) or expressive writing (Sayer et al., 2015); and for maladaptive coping styles (including avoidance and rumination), these can be addressed through traditional CBT techniques that often combined together in treatments such as the Unified Protocol (Barlow et al., 2017), which combines cognitive therapy, behavioural therapy and mindfulness (Farchione et al., 2012).

The findings from this study must be interpreted noting some methodological limitations, including the use of self-report data and use of abbreviated measures to accommodate the need for brevity of the assessment battery. Moreover, as is the case in many longitudinal studies, there were relatively high levels of attrition – however, regression analyses indicated few major differences across participants who were retained *v.* excluded from the sample, thus limiting evidence of systematic bias from study attrition. It should also be noted that due to the long time-course of the study, which began recruitment in 2009, the primary outcome for posttraumatic stress (the PCL-4) has more recently been updated and replaced with the PCL-5. Additionally, it must be recognized that the timing of when traumatic events occurred for participants was not specified and is therefore unknown.

With regards to the analyses, the conditional LGCA models were all bivariate, and did not control for variance that may be shared across explanatory variables. This approach was appropriate given the study was the first to consider the predictors of trajectory classes among early career military personnel, and there were no strong expectations regarding potential causal mechanisms including confounding and mediation (the latter of which may contraindicate multiple variable predictive models). However, it is important to highlight that the conditional analyses only indicate bivariate correlates of trajectory group membership, and potential causal mechanisms underlying these associations (including possible confounding) remain unclear.

The next steps building from the present study should examine the generalizability of these relationships across subpopulations divided by factors such as service branch, rank or gender – further examination of potential differences across service branches and how the factors which underpin mental health trajectories may differ accordingly would extend our understanding of how to tailor early identification and intervention for military members. This study has identified a key difference between service branches (Navy compared to Army) but this warrants further exploration.

This is the first study to explore the trajectories of key mental health outcomes, and factors associated with these trajectories, over the early years of the military career. The study provides a unique lens into data to inform ‘upstream’ early interventions to influence mental health trajectories and the potential mitigation of subsequent psychiatric disorder, the significant rates of which have been extensively documented in international research. Whilst most members adjusted to their new military career well, a portion demonstrated psychological maladjustment, indicating that the symptoms of mental health problems can begin to emerge as early in the military lifecycle. In the absence of preventative interventions, these military members'

## Supplementary material

For supplementary material accompanying this paper visit https://doi.org/10.1017/S0033291722000332. click here to view supplementary material
