# Supplementary material for: Mental health across the early years in the military
Source: Psychol Med. 2022 Feb 24;53(8):3683–91. doi: 10.1017/S0033291722000332 (PMC10277765; doi:10.1017/S0033291722000332)
Supplement: Supplementary file 1 [file S0033291722000332sup001.docx]

**Table S1.** Logistic regression models specifying T3 dropout as the dependent variable, with explanatory variables including sociodemographics and T2 levels of outcome variables.

|  | | **Odds Ratio** | **95% CI** |
| --- | --- | --- | --- |
| Age | | 0.96*** | 0.95 - 0.97 |
| Gender (ref. female) | |  |  |
|  | Male | 1.94*** | 1.66 - 2.27 |
| Relationship status (ref. single) | |  |  |
| Married/Cohabiting | | 0.74*** | 0.65 - 0.84 |
| Divorced/Separated/Widowed | | 0.92 | 0.54 - 1.66 |
| Number of children | | 0.86** | 0.79 - 0.94 |
| ADF Service (ref. Army) | |  |  |
| Navy | | 1.04 | 0.90 - 1.20 |
| Airforce | | 0.43*** | 0.37 - 0.51 |
| Posttraumatic stress (PCL-4) | | 1.00 | 0.96 - 1.02 |
| Psychological distress (K10) | | 1.01 | 0.99 - 1.02 |
| Anger (DAR-5) | | 1.02*** | 1.01 - 1.04 |
| Sleep problems (SII) | | 0.99 | 0.97 - 1.01 |

*Note*. CI = confidence interval. **p ≤ .01, ***p ≤ .001.

***Figure S2.*** *Flow-chart of the study data collection phases*.

*Note*. GE = General Enlistees.

**Table S3**. Eigenvalues from exploratory factor analysis (EFA) models and internal consistency estimates for all outcome measures across time.

| Variable | | Time point | Eigenvalues > 1.0 | | Ratio  [1:2] | Cronbach’s α (Pearson’s r) |
| --- | --- | --- | --- | --- | --- | --- |
|  |  |  | 1 | 2 |  |  |
| K10 | | T2 | 4.56 | 1.06 | 4.30 | 0.85 |
|  |  | T3 | 5.51 | 1.03 | 5.33 | 0.90 |
|  |  | T4 | 5.84 | 1.00 | 5.83 | 0.91 |
|  |  | T5 | 5.86 | 0.91 | 6.41 | 0.91 |
| PCL-4 | | T2 | 2.44 | 0.62 | 3.96 | 0.77 |
|  |  | T3 | 2.72 | 0.48 | 5.72 | 0.84 |
|  |  | T4 | 2.79 | 0.45 | 6.16 | 0.85 |
|  |  | T5 | 2.87 | 0.47 | 6.15 | 0.86 |
| DAR-5 | | T2 | 3.99 | 0.80 | 5.02 | 0.86 |
|  |  | T3 | 4.38 | 0.70 | 6.23 | 0.89 |
|  |  | T4 | 4.58 | 0.64 | 7.12 | 0.91 |
|  |  | T5 | 4.52 | 0.74 | 6.14 | 0.90 |
| SII | | T2 | 2.34 | 0.68 | 3.44 | 0.75 |
|  |  | T3 | 2.53 | 0.65 | 3.93 | 0.80 |
|  |  | T4 | 2.58 | 0.60 | 4.27 | 0.81 |
|  |  | T5 | 2.62 | 0.57 | 4.59 | 0.82 |
| Social Support | |  |  |  |  |  |
|  | Family/Friend Support | T2 | 2.16 | 1.20 | 1.80 | 0.71 |
|  |  | T3 | 2.30 | 1.18 | 1.94 | 0.75 |
|  |  | T4 | 2.54 | 0.99 | 2.56 | 0.81 |
|  |  | T5 | 2.53 | 1.06 | 2.39 | 0.81 |
|  | Family/Friend Negative Interactions | T2 | 2.25 | 0.89 | 1.13 | 0.74 |
|  |  | T3 | 2.27 | 0.94 | 1.13 | 0.74 |
|  |  | T4 | 2.34 | 0.87 | 1.17 | 0.76 |
|  |  | T5 | 2.37 | 0.88 | 1.19 | 0.77 |
|  | ADF Peer Support | T2 | — | — | — | (0.67) |
|  |  | T3 | — | — | — | (0.75) |
|  |  | T4 | — | — | — | — |
|  |  | T5 | — | — | — | — |
|  | ADF Peer Negative Interactions | T2 | 2.25 | 0.89 | 1.13 | 0.74 |
|  |  | T3 | 2.25 | 0.89 | 1.13 | 0.74 |
|  |  | T4 | — | — | — | — |
|  |  | T5 | — | — | — | — |
|  | ADF Superior Support | T2 | — | — | — | (0.69) |
|  |  | T3 | — | — | — | (0.76) |
|  |  | T4 | — | — | — | — |
|  |  | T5 | — | — | — | — |
|  | ADF Superior Negative Interactions | T2 | 2.25 | 0.89 | 1.13 | 0.74 |
|  |  | T3 | 2.25 | 0.89 | 1.13 | 0.74 |
|  |  | T4 | — | — | — | — |
|  |  | T5 | — | — | — | — |
| Coping* | |  |  |  |  |  |
| Support-seeking | | T2 | 3.01 | 0.48 | 6.27 | 0.89 |
|  | | T3 | 3.25 | 0.38 | 8.55 | 0.92 |
|  | | T4 | 3.25 | 0.35 | 9.29 | 0.92 |
|  | | T5 | 3.20 | 0.40 | 8.00 | 0.92 |
| Acceptance | | T2 | — | — | — | (0.65) |
|  | | T3 | — | — | — | (0.67) |
|  | | T4 | — | — | — | (0.70) |
|  | | T5 | — | — | — | (0.71) |
| Reappraisal | | T2 | — | — | — | (0.69) |
|  | | T3 | — | — | — | (0.76) |
|  | | T4 | — | — | — | (0.76) |
|  | | T5 | — | — | — | (0.74) |
| Self-blame | | T2 | — | — | — | (0.66) |
|  | | T3 | — | — | — | (0.68) |
|  | | T4 | — | — | — | (0.70) |
|  | | T5 | — | — | — | (0.72) |

Note. Pearson’s r correlations, denoted by (), were calculated in place of Cronbach’s alpha for scales with fewer than three items, or for measures which are not based on principles of reflective measurement which require assumptions of dimensionality or internal consistency (e.g. AUDIT-C). * the avoidance and risk-taking subscales were comprised of single item indicators and therefore Cronbach’s alpha is not relevant.

 
**Table S4.** Model fit indices for the K10 and PCL-4 plausible unconditional LCGAs

| **Model** | **Log likelihood** | **AIC** | **BIC** | **Entropy** | **LMR-LRT (p-value)** |
| --- | --- | --- | --- | --- | --- |
| K10 |  |  |  |  |  |
| 2-class | −27887.6 | 55797.3 | 55869.6 | 0.88 | 0.00 |
| 3-class | −27479.0 | 54988.0 | 55086.6 | 0.83 | 0.00 |
| 4-class | −27250.5 | 54538.9 | 54663.8 | 0.83 | 0.22 |
| PCL-4 |  |  |  |  |  |
| 2-class | −18142.8 | 36303.6 | 36362.7 | 0.98 | 0.00 |
| 3-class | −17303.5 | 34631.1 | 34709.8 | 0.96 | 0.00 |
| 4-class | −16885.6 | 33801.2 | 33899.7 | 0.89 | 0.00 |
| 5-class | −16550.9 | 33137.7 | 33255.9 | 0.90 | 0.40 |
| Note: AIC = Akaike’s Information Criterion; BIC = Bayesian Information Criterion; LMR-LRT = Lo-Mendell-Rubin Likelihood Ratio Test. | | | | | |

**Table S5.** The remaining conditional LCGA models with T2 predictors of class membership for the preferred 3-class model of K10 scores

|  | **Worsening vs Recovery** | | |  |
| --- | --- | --- | --- | --- |
|  | **Estimate** | **SE** | **Odds Ratio** | **95% CI** |
| Age | −0.01 | 0.02 | 0.99 | 0.95 - 1.04 |
| Gender (ref: Female) |  |  |  |  |
| Male | −0.02 | 0.26 | 0.98 | 0.59 - 1.64 |
| Relationship status (ref: single) |  |  |  |  |
| Partnered/DSW | 0.16 | 0.24 | 1.18 | 0.73 - 1.90 |
| Number of children | −0.03 | 0.18 | 0.97 | 0.68 - 1.39 |
| Rank (ref: GE) |  |  |  |  |
| Officer | −0.17 | 0.22 | 0.84 | 0.54 - 1.31 |
| ADF Service (ref: Army) |  |  |  |  |
| Navy | 0.13 | 0.25 | 1.14 | 0.69 - 1.87 |
| Airforce | 0.08 | 0.32 | 1.08 | 0.57 - 2.03 |
| Social support |  |  |  |  |
| Family/Friend social support | −0.08 | 0.05 | 0.92 | 0.84 - 1.02 |
| Family/Friend Negative Social Interactions | −0.01 | 0.05 | 0.09 | 0.91 - 1.09 |
| ADF social support |  |  |  |  |
| Peer Social Support | −0.19 | 0.08 | 0.83* | 0.71 - 0.96 |
| ADF Peer Negative Social Interactions | 0.20 | 0.06 | 1.22** | 1.08 - 1.37 |
| Superior Social Support | −0.24 | 0.07 | 0.79** | 0.69 - 0.90 |
| ADF Superior Negative Social Interactions | 0.20 | 0.05 | 1.23*** | 1.11 - 1.35 |
| Number of traumatic events | 0.04 | 0.04 | 1.04 | 0.96 - 1.11 |
| Coping styles |  |  |  |  |
| Acceptance | −0.45 | 0.08 | 0.64*** | 0.55 - 0.75 |
| Reappraisal | −0.33 | 0.08 | 0.72*** | 0.62 - 0.84 |
| Self-blame | 0.33 | 0.06 | 1.39*** | 1.23 - 1.57 |
| Avoidance | 0.37 | 0.12 | 1.44*** | 1.15 - 1.81 |
| Risk-taking | 0.28 | 0.13 | 1.32* | 1.03 - 1.70 |
| Support-seeking | −0.01 | 0.04 | 0.99 | 0.91 - 1.07 |
| Anger | 0.06 | 0.02 | 1.06** | 1.02 - 1.09 |
| Sleep problems | 0.17 | 0.03 | 1.19*** | 1.12 - 1.27 |

*Note*. CI = confidence interval. *p ≤ .05, **p ≤ .01, ***p ≤ .001.

**Table S6.** The remaining conditional LCGA models with T2 predictors of class membership for the preferred 4-class model of PCL-4 scores

|  | **Chronic-subthreshold vs Worsening** | | | | **Chronic-subthreshold vs Recovery** | | | | **Worsening vs Recovery** | | | | | |  |  |
| --- | --- | --- | --- | --- | --- | --- | --- | --- | --- | --- | --- | --- | --- | --- | --- | --- |
|  | **Estimate** | **SE** | **Odds Ratio** | **95% CI** | **Estimate** | **SE** | **Odds Ratio** | **95% CI** |  | | **Estimate** | **SE** | **Odds Ratio** | **95% CI** | |  |
| Age | 0.06 | 0.02 | 1.06** | 1.02 - 1.10 | 0.02 | 0.02 | 1.02 | 0.98 - 1.06 | | −0.04 | | 0.02 | 0.96 | 0.92 - 1.00 | | |
| Gender (ref: Female) |  |  |  |  |  |  |  |  | |  | |  |  |  | | |
| Male | −0.70 | 0.25 | 0.50** | 0.31 - 0.80 | −0.30 | 0.26 | 0.74 | 0.45 - 1.22 | | 0.40 | | 0.30 | 1.49 | 0.83 - 2.70 | | |
| Relationship status (ref: single) |  |  |  |  |  |  |  |  | |  | |  |  |  | | |
| Partnered/DSW | 0.24 | 0.23 | 1.27 | 0.80 - 2.01 | −0.02 | 0.23 | 0.99 | 0.62 - 1.56 | | −0.26 | | 0.29 | 0.77 | 0.44 - 1.36 | | |
| Number of children | 0.14 | 0.16 | 1.15 | 0.83 - 1.59 | 0.06 | 0.17 | 1.06 | 0.76 - 1.49 | | −0.08 | | 0.20 | 0.92 | 0.63 - 1.36 | | |
| Rank (ref: GE) |  |  |  |  |  |  |  |  | |  | |  |  |  | | |
| Officer | −0.14 | 0.22 | 0.87 | 0.57 - 1.33 | −0.32 | 0.21 | 0.73 | 0.48 - 1.10 | | −0.18 | | 0.27 | 0.84 | 0.50 - 1.41 | | |
| ADF Service (ref: Army) |  |  |  |  |  |  |  |  | |  | |  |  |  | | |
| Navy | 0.34 | 0.25 | 1.40 | 0.86 - 2.30 | 0.23 | 0.24 | 1.26 | 0.79 - 2.00 | | −0.11 | | 0.30 | 0.90 | 0.50 - 1.62 | | |
| Airforce | 0.44 | 0.30 | 1.56 | 0.86 - 2.83 | −0.20 | 0.34 | 0.82 | 0.42 - 1.60 | | −0.64 | | 0.40 | 0.53 | 0.24 - 1.16 | | |
| Social support |  |  |  |  |  |  |  |  | |  | |  |  |  | | |
| Family/Friend social support | 0.08 | 0.06 | 1.08 | 0.95 - 1.23 | −0.19 | 0.05 | 0.83*** | 0.76 - 0.91 | | −0.26 | | 0.07 | 0.77*** | 0.67 - 0.89 | | |
| ADF social support |  |  |  |  |  |  |  |  | |  | |  |  |  | | |
| Peer Social Support | 0.09 | 0.08 | 1.10 | 0.94 - 1.29 | −0.34 | 0.08 | 0.71*** | 0.61 - 0.83 | | −0.43 | | 0.10 | 0.65*** | 0.54 - 0.79 | | |
| Superior Social Support | 0.21 | 0.08 | 1.23** | 1.06 - 1.43 | −0.20 | 0.07 | 0.82** | 0.72 - 0.94 | | −0.41 | | 0.09 | 0.66*** | 0.56 - 0.79 | | |
| Negative Social Interactions |  |  |  |  |  |  |  |  | |  | |  |  |  | | |
| Family/Friend | −0.16 | 0.04 | 0.85*** | 0.78 - 0.93 | 0.08 | 0.04 | 1.09* | 1.00 - 1.18 | | 0.24 | | 0.05 | 1.27*** | 1.14 - 1.41 | | |
| ADF peers | −0.16 | 0.06 | 0.85** | 0.76 - 0.96 | 0.15 | 0.05 | 1.16** | 1.04 - 1.29 | | 0.31 | | 0.07 | 1.36*** | 1.18 - 1.57 | | |
| ADF superiors | −0.16 | 0.05 | 0.85** | 0.77 - 0.94 | 0.16 | 0.05 | 1.17** | 1.06 - 1.29 | | 0.32 | | 0.06 | 1.38*** | 1.22 - 1.56 | | |
| Number of traumatic events | 0.01 | 0.04 | 1.01 | 0.94 - 1.09 | 0.13 | 0.03 | 1.14*** | 1.07 - 1.21 | | 0.12 | | 0.04 | 1.13** | 1.03 - 1.22 | | |
| Coping styles |  |  |  |  |  |  |  |  | |  | |  |  |  | | |
| Acceptance | 0.31 | 0.09 | 1.37** | 1.14 - 1.64 | −0.23 | 0.08 | 0.79** | 0.68 - 0.93 | | −0.54 | | 0.11 | 0.58*** | 0.47 - 0.72 | | |
| Reappraisal | 0.32 | 0.08 | 1.38*** | 1.19 - 1.60 | −0.10 | 0.07 | 0.90 | 0.78 - 1.04 | | −0.43 | | 0.09 | 0.65*** | 0.54 - 0.78 | | |
| Self-blame | −0.29 | 0.07 | 0.75*** | 0.65 - 0.86 | 0.33 | 0.06 | 1.39*** | 1.23 - 1.58 | | 0.62 | | 0.09 | 1.86*** | 1.57 - 2.19 | | |
| Avoidance | −0.61 | 0.06 | 0.55*** | 0.49 - 0.61 | 0.27 | 0.12 | 1.31* | 1.04 - 1.66 | | 0.88 | | 0.16 | 2.41*** | 1.75 - 3.32 | | |
| Risk-taking | −0.21 | 0.15 | 0.81 | 0.60 - 1.09 | 0.34 | 0.11 | 1.41** | 1.14 - 1.75 | | 0.55 | | 0.17 | 1.74** | 1.26 - 2.40 | | |
| Support-seeking | 0.07 | 0.04 | 1.08 | 1.00 - 1.16 | 0.00 | 0.04 | 1.00 | 0.92 - 1.08 | | −0.08 | | 0.05 | 0.93 | 0.84 - 1.02 | | |
| Anger | −0.11 | 0.03 | 0.90*** | 0.86 - 0.94 | 0.08 | 0.01 | 1.08*** | 1.05 - 1.11 | | 0.19 | | 0.03 | 1.21*** | 1.14 - 1.27 | | |
| Sleep problems | −0.21 | 0.04 | 0.81*** | 0.75 - 0.87 | 0.13 | 0.03 | 1.14*** | 1.08 - 1.20 | | 0.34 | | 0.04 | 1.40*** | 1.29 - 1.53 | | |

*Note*. CI = confidence interval. *p ≤ .05, **p ≤ .01, ***p ≤ .001
